# Associations of Word Memory, Verbal Fluency, Processing Speed, and Crystallized Cognitive Ability With One-Legged Balance Performance in Mid- and Later Life

**DOI:** 10.1093/gerona/glab168

**Published:** 2021-06-14

**Authors:** Joanna M Blodgett, Rachel Cooper, Daniel H J Davis, Diana Kuh, Rebecca Hardy

**Affiliations:** 1 MRC Unit for Lifelong Health and Ageing at UCL, London, UK; 2 Musculoskeletal Science and Sports Medicine Research Centre, Department of Sport and Exercise Sciences, Manchester Metropolitan University, UK; 3 CLOSER, Social Research Institute, UCL, London, UK

**Keywords:** Birth cohort, Cognitive aging, Epidemiology, Life course, Physical performance

## Abstract

**Background:**

Cognitive integration of sensory input and motor output plays an important role in balance. Despite this, it is not clear if specific cognitive processes are associated with balance and how these associations change with age. We examined longitudinal associations of word memory, verbal fluency, search speed, and reading ability with repeated measures of one-legged balance performance.

**Method:**

Up to 2 934 participants in the MRC National Survey of Health and Development, a British birth cohort study, were included. At age 53, word memory, verbal fluency, search speed, and reading ability were assessed. One-legged balance times (eyes closed) were measured at ages 53, 60–64, and 69 years. Associations between each cognitive measure and balance time were assessed using random-effects models. Adjustments were made for sex, death, attrition, height, body mass index, health conditions, health behaviors, education, and occupational class.

**Results:**

In sex-adjusted models, 1 *SD* higher scores in word memory, search speed, and verbal fluency were associated with 14.1% (95% CI: 11.3, 16.8), 7.2% (4.4, 9.9), and 10.3% (7.5, 13.0) better balance times at age 53, respectively. Higher reading scores were associated with better balance, although this association plateaued. Associations were partially attenuated in mutually adjusted models and effect sizes were smaller at ages 60–64 and 69. In fully adjusted models, associations were largely explained by education, although remained for word memory and search speed.

**Conclusions:**

Higher cognitive performance across all measures was independently associated with better balance performance in midlife. Identification of individual cognitive mechanisms involved in balance could lead to opportunities for targeted interventions in midlife.

Age-related declines in cognitive and physical capability from midlife onwards are common, with increasing recognition of the interdependency of these 2 domains; this includes theories of an underlying common cause of aging (1) and evidence that declines in one domain may contribute directly to declines in the other (2–6). Emerging evidence suggests that performance on cognitive tests may provide early indications of changes in physical capability (6–9). Balance is one of the physical capability measures most closely linked to cognitive ability, given the crucial neural integration of sensory input and motor response required to maintain balance (10). Recent analyses from our group provided evidence of an association between midlife word memory and subsequent balance performance and identified research gaps which need addressing to further understand this association (5).

Although better overall cognition is associated with better balance performance (3,11–14), the role of specific cortical processes in maintaining balance is not well established. Given the complexity of neural circuits involved in balance and gait tasks (10), specific cognitive processes may play independent roles in maintaining balance. For example, poor functioning in areas of the brain responsible for memory, executive function, or processing speed could partially explain poor balance (10,15,16). Existing evidence suggests that there are conflicting patterns of association between different cognitive processes and balance performance (12–14,17), lending support to the involvement of distinct cognitive pathways.

Most evidence on individual cognitive measures (eg, memory, subcomponents of executive function) and balance has relied on cross-sectional data, small sample sizes, and/or older, age-heterogeneous samples (≥65 years) (3,11–14). Furthermore, studies have not investigated if specific cognitive processes have independent roles in maintaining balance nor assessed alternate pathways that may explain the association such as socioeconomic position (SEP), health status, or health behaviors.

Previous findings from the MRC National Survey of Health and Development (NSHD) have identified positive associations between general cognitive performance in childhood and one-legged balance performance over 3 time points in midlife (18) and between individual cognitive measures in adulthood with balance at age 53 (17). In our most recent analyses, we found an association between word memory and subsequent balance performance, but no evidence of association in the opposite direction (ie, balance performance and subsequent word memory). This association weakened between ages 53 and 69 years and other cognitive measures were not examined (5). Investigating longitudinal cognitive–balance associations across multiple cognitive measures could have important implications for understanding age-related decline in balance performance. For example, single cognitive tests could identify individuals at risk of poor balance, providing opportunities for both screening and interventions. Understanding these associations earlier in midlife, either before or in the early stages of decline, is particularly important to prevent or mitigate loss of independent mobility.

To address important limitations within the current evidence base, our primary aim was to examine longitudinal associations between 4 cognitive measures (word memory, verbal fluency, processing speed, and crystallized cognitive ability) at age 53 and one-legged balance performance at ages 53, 60–64, and 69. Given limited and conflicting evidence in this area, we also sought to test if these associations differed by (i) age using repeated balance assessments or (ii) sex, and if they remained after (iii) mutual adjustment for other cognitive measures and (iv) adjustment for education, other indicators of SEP, health status, and health behaviors. We hypothesized that higher cognitive performance on all tests would be associated with better balance performance at all ages. We expected associations with crystallized cognitive ability to attenuate after adjustment for other cognitive measures, but to remain for the fluid cognitive measures (eg, memory, verbal fluency, processing speed). Finally, we expected associations to weaken after adjustment for SEP, health status, and health behaviors.

## Methods

### Study Sample

**Figure 1.**
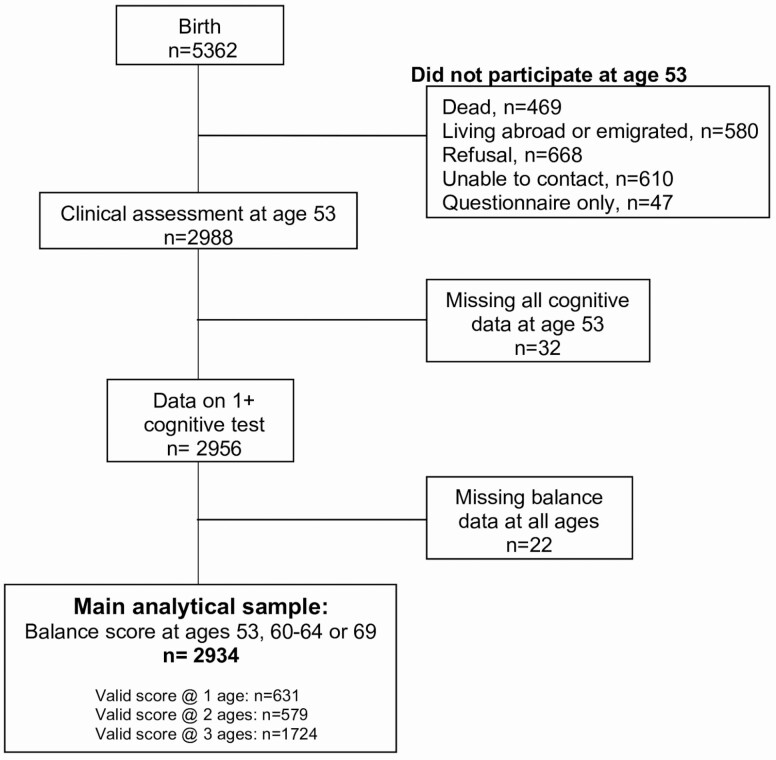
Derivation of analytical sample in MRC National Survey of Health and Development.

Data from the MRC NSHD were used. NSHD is a birth cohort of 5 362 individuals born in England, Scotland, and Wales in 1 week in March 1946. Study members have been followed up to 24 times from birth to the most recent data collection in 2015 (age 69). The cohort profile, participation rates, and characteristics of those lost to follow-up have been previously detailed (19–21). As the main focus of the analyses is on cognitive measures at age 53, we considered participants who had participated in a nurse home visit at this age. By age 53, 469 of the original cohort had died, 580 had emigrated or were temporarily living abroad, 610 were unable to be traced, 668 refused to participate, and 47 responded to a questionnaire only. Of the 2 988 study members who participated at age 53, 32 (1.1%) individuals were missing cognitive data at this age and 22 (0.7%) had no balance data at any age. The final analytical sample (*n* = 2 934) consisted of those who had cognitive data at age 53 and at least one balance score at ages 53, 60–64, or 69 (see Figure 1).

### Assessment of Balance and Cognition

*One-legged balance performance* was assessed at ages 53, 60–64, and 69 using a standardized protocol to assess static balance and postural control. Individuals were instructed by a research nurse to cross their arms, close their eyes, and stand on one leg and maintain this position for as long as possible up to a maximum of 30 seconds. Time was recorded in seconds at age 53 and to the nearest 0.01 second at ages 60–64 and 69. Timing stopped when the suspended leg touched the ground or after the maximum time was reached. If participants fell over straight away or were unable to complete the test due to health reasons, they were allocated a score of 0 for the purpose of these analyses (*n* = 335 scores in 277 individuals). The one-legged balance test is a reliable measure of balance, with high interrater and test–retest reliability (22–27).

*Short-term memory* was assessed at age 53 using a *word memory* recall task. Participants were presented with 15 words at a rate of 1 word every 2 seconds. They were then immediately asked to write down all of the words that they could recall. Three trials were administered with the total score representing the number of words correctly recalled across all trials (maximum score: 45).

*Verbal fluency*, a component of executive function, was assessed at age 53 using an animal naming task, in which study members were asked to name as many animals as possible within 1 minute. Species (eg, bird, snowy owl, blue jay, etc.), sex, and generation-specific names (eg, bull, cow, calf) were considered as different names, while repetitions and redundancies (eg, black cow, brown cow) were not. The score was the number of different animals named.

*Processing speed* was assessed at age 53 using a visual *search speed* task. Study members were given a grid of letters and, starting at the top left corner of the grid, were asked to go through each line and cross out as many “P’s” and “W’s” as they could, as quickly and as accurately as possible, in 1 minute. The score represents the total number of letters searched (maximum: 600).

*Crystallized cognitive ability* was assessed at age 53 using the National Adult Reading Test (NART), a test of general verbal knowledge that is commonly used to assess premorbid intelligence levels (28). Study members were asked to pronounce a set of 50 words and responses were scored as correct or incorrect (maximum score: 50). These words are all atypical in grapheme–phoneme correspondence, such that they are only likely to be pronounced correctly if the study member has previous knowledge of the word. Examples include “hiatus,” “syncope,” and “placebo.”

The psychometric properties of all 4 cognitive tests have been extensively investigated and described elsewhere. In both healthy community-dwelling samples and those with cognitive impairment, all tests have high interrater (.90–.98) and test–retest reliability (.68–.98) (29–32). 

### Assessment of Covariates

Health status, health behaviors, height, and body mass index (BMI) were each assessed at ages 53, 60–64, and 69 and thus were treated as time-varying covariates. Measures of health status included binary indicators of *diabetes*, *knee pain*, history of *cardiovascular events* and *respiratory symptoms*, and a continuous measure of *symptoms of depression and anxiety* (28-item General Health Questionnaire; range: 0–84). Health behaviors included self-reported *leisure time physical activity* (never, 1–4 times/mo, 5+ times/mo) and *smoking status* (never, past smoker, current smoker). *Height* (cm) and *BMI* (kg/m^2^; derived from height and weight measurements) were ascertained by research nurses. *Adult occupational class* was recorded at age 53 using the Registrar General’s Social Classification (I Professional/II Intermediate, III Skilled nonmanual or manual, IV Partly skilled/V Unskilled manual). Highest level of *education*al *attainment* (degree or higher; advanced secondary qualifications generally attained at 18 years; ordinary secondary qualifications generally attained at 16 years; below ordinary secondary qualifications; none) was self-reported at age 26.

### Statistical Analyses

Mann–Whitney *U* and *t* tests were used to examine sex differences in median (interquartile range) balance and mean (*SD*) cognitive scores. Pearson’s correlation coefficients assessed correlations between scores on each of the different cognitive tests. Due to its right skew, balance times were log-transformed for all regression analyses and all estimates are presented as sympercents (% change) (33). One second was added to all balance times prior to log-transformation to circumvent log values of zero.

Associations between each cognitive score at age 53 and balance performance at ages 53, 60–64, and 69 were assessed with random-effects models which account for repeated balance scores at 3 different ages nested within individuals. The intercept represents balance performance at age 53, with the intercept and the slope modeled as random effects. Age-by-cognitive test interaction terms were included to assess if the associations between cognitive scores and balance performance changed with age. Interactions between sex and each cognitive measure were also assessed and models were stratified by men and women where significant (at *p* < .05). Quadratic cognitive terms were tested for all scores (also at *p* < .05) to check for evidence of deviation from linearity. First, sex-adjusted (or stratified) models were used to investigate the association between cognition at age 53 (word memory, search speed, verbal fluency, and NART) and balance performance in 4 individual models. Next, all cognitive scores were included in a mutually adjusted model to assess independent associations with balance performance. Finally, covariates were added in 5 stages (anthropometric measures, health status, health behaviors, adult occupational class, education), with a final model that was fully adjusted for all covariates. At each stage, quadratic covariate terms, sex by covariate, and age by covariate interactions were included where significant at *p* <.05. Binary indicators of death and attrition between ages 53 and 69 were included in these analyses to minimize bias that may have been introduced by loss to follow-up in those with poorer balance performance (21,34).

Characteristics of those with missing cognitive scores or covariate data at age 53 were compared with the main analytical sample and cognitive scores were compared between individuals with missing balance data at any age. Sensitivity analyses replicated the initial sex-adjusted (or stratified) models using the maximal available sample for each cognitive test. Sensitivity analyses were also undertaken in the final fully adjusted model, where nonsignificant Age × Cognitive terms were removed from the model. All analyses were conducted in Stata 15.1.

## Results

### Descriptive Characteristics

Median (Q1, Q3) balance times and mean (*SD*) cognitive scores are shown in Table 1. Balance times were heavily right-skewed (5); 60%, 74%, and 80% of individuals had a balance time between 0 and 5 seconds at ages 53, 60–64, and 69, respectively. Balance times declined with age in both men and women, although men had better balance times than women at all ages (*p* < .001 for all ages). Women performed better than men on the visual search speed and word memory tests (both *p* < .001). There were no sex differences in verbal fluency (*p* = .3) or NART scores (*p* = .7). In men and women, NART and word memory scores were the 2 most highly correlated tests (*r* = .53), while NART and search speed scores were the least strongly correlated (*r* = .15). Detailed characteristics of a larger sample from NSHD have been described elsewhere (35); the characteristics at age 53 in men and women are described in Supplementary Table 1.

**Table 1.** Mean Balance Performance and Cognitive Test Scores by Sex at Age 53 in Maximum Available Sample (up to *n* = 2 934)

	Men	Women	Tests of Sex Difference (*p* value)
Balance time (eyes closed) (s)			
Age 53 y, *n*	1 405	1 468	<.001^a^
Median (Q1, Q3)	5 (3, 10)	4 (3, 7)	
Age 60–64 y, *n*	982	1 107	<.001^a^
Median (Q1, Q3)	3.57 (2.34, 5.50)	3.17 (2.16, 4.78)	
Age 69 y, *n*	956	1 043	<.001^a^
Median (Q1, Q3)	2.99 (1.89, 4.88)	2.72 (1.67, 4.15)	
Range at all ages (min–max)	0–30	0–30	
Word memory, *n*	1 367	1 442	
Mean (*SD*)	23.0 (6.2)	24.9 (6.2)	<.001^b^
Range	4–40	3–41	
Search speed, *n*	1 396	1 454	
Mean (*SD*)	272.9 (75.7)	289.9 (75.6)	<.001^b^
Range	91–591	64–591	
Verbal fluency, *n*	1 400	1 466	
Mean (*SD*)	23.8 (6.7)	23.5 (7.1)	.27^b^
Range	1–47	1–62	
NART, *n*	1 330	1 419	
Mean (*SD*)	34.5 (9.6)	34.3 (9.4)	.72^b^
Range	2–50	1–50	

*Notes*: NART = National Adult Reading Test; Q1 = quartile 1; Q3 = quartile 3.

^a^Mann–Whitney *U* test.

^b^ *t*-Test for comparison of mean (*SD*).

### Individual Sex-Adjusted Models (Models 1–4)

**Figure 2.**
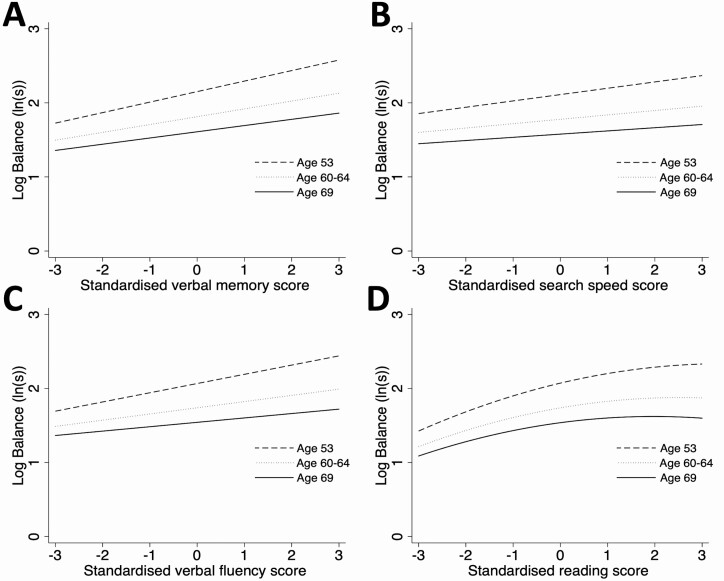
Sex-adjusted models showing associations between cognitive score and balance performance at ages 53, 60–64, and 69 (*n* = 2 549, obs = 5 466) for (**A**) word memory, (**B**) search speed, (**C**) verbal fluency, and (**D**) NART scores. NART = National Adult Reading Test.

In random-effects models, 2 549 individuals had complete balance and covariate data at one or more ages, contributing 5 466 balance observations. There was no evidence of sex differences in associations between any of the cognitive tests and balance time (*p* > .15). In sex-adjusted models containing each cognitive score separately, 1 *SD* higher scores in word memory, search speed, and verbal fluency were associated with 14.1% (95% CI: 11.3, 16.8), 7.2% (4.4, 9.9), and 10.3% (7.5, 13.0) better balance times at age 53, respectively (Table 2, Models 1–3; Figure 2A–C). NART scores demonstrated a quadratic association with balance performance, such that better scores were associated with better balance but this effect plateaued above a certain score (Table 2, Model 4; Figure 2D).

**Table 2.** Random-Effects Models Assessing Longitudinal Associations of Each of 4 Cognitive Test Scores at Age 53 With Log-Transformed Balance Time (ln(s)) at Ages 53–69 (*n* = 2 549 [5 466]; complete cases)

	% Change in Association per 1 y		Mean % Difference in Balance Time (s) per 1 *SD* Change in Cognition at:					
			Age 53		Age 60–64		Age 69	
	Age × Cognition Coefficient (95% CI)	*p* Value	Coefficient (95% CI)	*p* Value	Coefficient (95% CI)	*p* Value	Coefficient (95% CI)	*p* Value
Sex-adjusted Models 1–4								
1. Word memory	−0.49 (−0.73, −0.25)	<.001	14.1 (11.3, 16.8)	<.001	9.2 (7.0, 1.4)	<.001	6.2 (3.3, 9.2)	<.001
2. Search speed	−0.20 (−0.44, 0.04)	.10	7.2 (4.4, 9.9)	<.001	5.2 (3.0, 7.3)	<.001	4.0 (1.1, 6.9)	<.01
3. Verbal fluency	−0.39 (−0.63, −0.15)	<.001	10.3 (7.5, 13.0)	<.001	6.4 (4.2, 8.5)	<.001	4.0 (1.1, 6.9)	<.01
4. NART								
Linear term	−0.49 (−0.73, −0.25)	<.001	14.7 (11.8, 17.7)	<.001	9.8 (7.4, 12.2)	<.001	6.9 (3.8, 10.0)	<.001
Quadratic term	—^a^		2.2 (0.6, 3.9)	<.01	Estimate constant^f^			
Mutually adjusted Model 5^b^								
1. Word memory	−0.32 (−0.60, −0.03)	.03	8.3 (5.0, 11.5)	<.001	5.1 (2.5, 7.7)	<.001	3.2 (−0.3, 6.7)	.07
2. Search speed	—^c^		3.4 (1.3, 5.5)	<.005	Estimate constant^f^			
3. Verbal fluency	—^c^		2.9 (0.7, 5.1)	.01	Estimate constant^f^			
4. NART								
Linear term	−0.32 (−0.60, −0.03)	.03	8.7 (5.3, 12.1)	<.001	5.5 (2.7, 8.3)	<.001	3.5 (−0.1, 7.2)	.06
Quadratic term	—^c^		1.8 (0.1, 3.5)	.03	Estimate constant^f^			
Fully adjusted Model 6^d^								
1. Word memory	−0.24 (−0.53, 0.05)	.11 ^e^	5.0 (1.7, 8.3)	<.005	2.6 (0.04, 5.2)	.05	1.2 (−2.3, 4.7)	.51
2. Search speed	—		2.2 (0.2, 4.1)	.03	Estimate constant^f^			
3. Verbal fluency	—		2.1 (−0.1, 4.2)	0.06	Estimate constant^f^			
4. NART								
Linear term	−0.23 (−0.54, 0.09)	.16 ^e^	3.3 (−0.4, 7.1)	.08	1.1 (−2.0, 4.2)	.48	−2.6 (−4.2, 3.7)	.90
Quadratic term	—		0.6 (−1.0, 2.3)	.46	Estimate constant^f^			

*Notes*: NART = National Adult Reading Test.

^a^Interaction term for Age × NART score^2^ (*p* = .95) was not significant and thus removed from the model.

^b^Adjusted for sex, Sex × Age, and all 3 other cognitive test scores and relevant interaction terms.

^c^Interaction terms for Age × Search speed (*p* = .50), Age × Verbal fluency (*p* = .19), and Age × NART^2^ (*p* = .88) were not significant and thus removed from the model.

^d^Adjusted for sex, Sex × Age, death, Death × Age × Sex, attrition, height, height^2^, body mass index, cardiovascular disease events, diabetes, respiratory symptoms, knee pain, Knee pain × Age, symptoms of depression and anxiety, Depression/anxiety × Age, smoking history, leisure time physical activity, Physical activity × Age, adult social class, education, and Education × Age.

^e^See sensitivity analyses in Supplementary Table 1 for models without Age × word memory and Age × NART interaction terms.

^f^Estimates at ages 60–64 and 69 are equivalent to those estimated at age 53.

For all cognitive scores, the association decreased with age as illustrated by the weaker estimated association between cognitive score and balance at later ages in Figure 2. For example, 1 *SD* increase in word memory was associated with 9.2% (7.0, 1.4) better balance performance at age 60–64, decreasing further to 6.2% (3.3, 9.2) at age 69 (Table 2, Model 1; Figure 2A). Similar changes in associations with age were observed for both search speed (Model 2, Figure 2B) and verbal fluency (Model 3, Figure 2C). The linear term for NART scores also decreased with age; however, the quadratic term remained constant (*p* = .95 for Age × NART^2^ interaction term); this suggests the association plateaued at lower scores with increasing age (Model 4, Figure 2D).

### Adjusted Models (Models 5 + 6)

**Figure 3.**
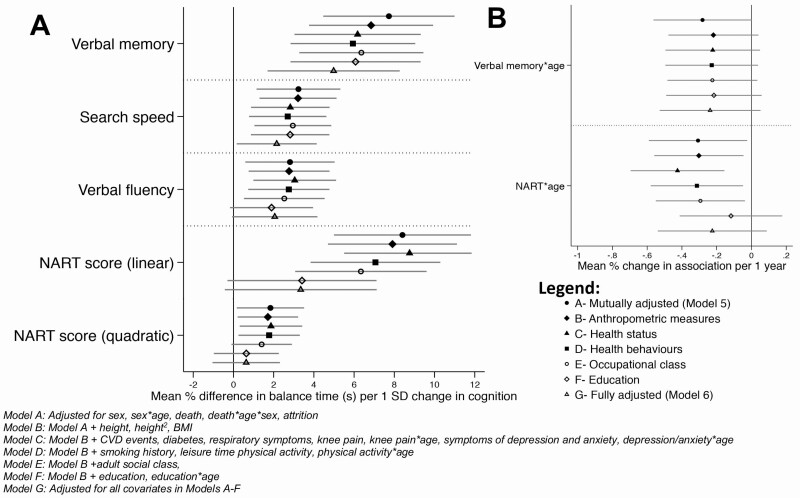
(**A**) Mean percent difference in balance time for all cognitive scores at each stage of adjustment and (**B**) Cognitive × Age interaction terms indicating the decreasing association of both word memory and NART with balance score over time. NART = National Adult Reading Test.

In a mutually adjusted model of all cognitive tests, estimates for each measure were attenuated by nearly half, although all cognitive scores remained associated with balance performance (Table 2, Model 5). Attenuation of age interaction terms suggests that associations for search speed and verbal fluency no longer became smaller at older ages. Figure 3 outlines the impact of adjustment for covariates at each stage (eg, B. anthropometric measures, C. health status indicators, D. health behaviors, E. occupational class, F. education), demonstrating that associations between most cognitive tests and balance performance remained. Notably, adjustment for education largely explained the smaller NART coefficients and NART by age interaction terms.

In the fully adjusted model, 1 *SD* increases in search speed and verbal fluency at age 53 were associated with 2.2% (0.2, 4.1) and 2.1% (−0.1, 4.2) better balance performance at all ages (53, 60–64, 69 years) (Table 2, Model 6). One *SD* increase in word memory was associated with 5.0% (1.7, 8.3) and 2.6% (0.04, 5.2) better balance performances at ages 53 and 60–64, although was no longer associated by age 69. Finally, the association between NART and balance performance was weak in fully adjusted models and no longer present at ages 60–64 or 69.

### Sensitivity Analyses

Compared with the main analytical sample (*n* = 2 934), those with missing cognitive data (*n* = 32) had worse balance performance at age 53 (*p* < .001) and those with missing balance data at all ages (*n* = 22) performed worse on all 4 cognitive tests (*p* < .05 for all). Individuals with complete covariate data (*n* = 2 445) performed better on the balance, word memory, and verbal fluency tests than those missing one or more covariates (*n* = 429), although there were no differences in search speed (*p* = .15) or NART scores (*p* = .18).

In separate cognitive models, associations did not change when examined in the maximal available sample (Supplementary Table 2, Models 1–4) compared with the complete cases (as presented in Table 2). Sensitivity analyses in the final model removed both NART × Age and Word memory × Age interaction terms and then each term in turn; results demonstrated that each interaction was stronger when only one was included (Supplementary Table 2, Models A–C). This contrasted with the final fully adjusted model, which produced weaker evidence that associations attenuated with age.

## Discussion

Higher cognitive performance in all 4 tests in midlife was associated with better one-legged balance performance. Associations were strongest cross-sectionally at age 53 and became smaller with increasing age. Changes in the associations of search speed and verbal fluency with balance by age were considerably attenuated after adjustment for covariates, while associations of word memory and reading scores with balance performance continued to weaken over time. Estimates were attenuated by approximately half when all cognitive scores were included in the model. Fully adjusted models suggested that memory, verbal fluency, and processing speed were associated with balance performance independent of each other and of all covariates, while associations between NART scores and balance were largely explained by educational attainment.

### Comparison With Other Studies

No previous study has investigated age-related changes in associations between specific cognitive processes and balance in midlife or considered mutual adjustment of multiple cognitive measures. As such, several of the study findings are novel and comparisons with other studies are limited. However, findings are generally consistent with evidence showing that individuals with higher overall cognitive ability have better balance performance (12,14,36,37). Fewer studies have examined specific cognitive measures, with mainly cross-sectional studies demonstrating different patterns of associations with balance across different processes (12–14,38).

Associations between higher scores on both processing speed and verbal fluency tasks and better balance performance are consistent with other studies (2,12–14,39). Conversely, the results for word memory are inconsistent with existing evidence. Although previous analyses of cross-sectional or lagged associations between memory and balance at ages 43 and 53 in NSHD are consistent with findings shown here (5,17), other studies have either reported no associations (2) or found evidence of an association for visual but not word memory (13). Only one non-NSHD study has examined crystallized cognition (measured with the Wechsler Adult Intelligence Scale-III), reporting no association with balance performance (13). This is consistent with our findings that suggest that fluid cognitive measures may be more strongly associated with balance than crystallized measures.

### Explanation of Possible Indirect and Direct Pathways

Differences in patterns of association support the hypothesis of direct and indirect pathways between cognition and balance. Attenuation of associations between NART (eg, crystallized cognitive ability) and balance, particularly at later ages, suggests an indirect pathway that may largely act via educational attainment. Intelligence, education, and SEP are all highly correlated; those with higher overall cognitive ability are more likely to have higher educational attainment and a higher SEP, and vice versa (40,41). These individuals are also more likely to partake in healthy behaviors, have positive health outcomes, better psychosocial support, and fewer physical impairments, including poorer balance performance (35, 42–44). Taken together, individuals with higher general cognitive ability may be better positioned to improve their health and maintain independent functioning (45). This is consistent with evidence suggesting that positive associations between childhood cognitive ability and balance performance in later life (18,46) are explained by socioeconomic, health and behavioral pathways (18).

Independent associations between all fluid cognitive measures—memory, processing speed, and verbal fluency—suggests that balance relies on the integration of multiple cognitive processes. *Short-term memory* draws on a temporary storage system (47), which may allow selective utilization of cognitive resources needed to maintain postural stability (48). Additionally, some memory tasks heavily rely on attention, which can play a large role in maintaining balance, depending on the complexity of the task. For example, selective attention can be important to filter out unimportant stimulus information (eg, distracting visual cues), while divided attention allows an individual to carry out more than one task at a time (eg, walking and talking) (49). Multiple dual-task paradigms, that include simultaneous physical and cognitive tasks, have demonstrated the importance of attention in balance (15,50). Differing contribution of attention to memory tasks could explain different patterns of association with balance performance (5,13,17,51).

Processing speed captures an underlying cognitive process that has an impact on the efficiency of all other cognitive operations (52). Slower processing speed is linked to reduced activity in the prefrontal cortex and dopamine deficiency, both of which are thought to impact balance and motor abilities (53–56). Individuals who are able to quickly process, react, and provide an integrated motor response may demonstrate better balance abilities. Finally, several subcomponents of executive function involved in balance may explain positive associations between *verbal fluency* and balance performance. For example, clustering and self-initiation occur when study participants generate a series of words that adhere to a specific, pre-existing theme (eg, first naming types of birds, then fish, etc.). Task switching occurs when subjects exhaust one theme and must switch their memory targets to a different cluster. Finally, action inhibition plays an important role in suppressing the tendency to repeat previously named animals or non-task specific words (57,58). Evidence from dual-task paradigms has shown that older adults are less able to divide their attention and memory between multiple tasks than younger adults (59); this could translate to difficulties maintaining balance in situations with competing cues.

### Understanding Changes With Age

Associations between all cognitive measures and balance performance became weaker with increasing age in individual models. The relative contribution of cognitive mechanisms may decrease as more proximal physical factors associated with aging such as visual impairment, muscular atrophy, and reduced vestibular functioning emerge. Previous NSHD research has shown that associations between knee pain and symptoms of depression and anxiety and balance ability grew stronger at older ages (35). Once all time-varying covariates, including health status indicators, were accounted for, associations between cognition and balance largely remained constant with age (as shown in Supplementary Table 2). This may suggest that the contribution of cognition to balance is similar throughout midlife. Further investigation of these associations in later life could allow better understanding of both mechanisms and implications of changing associations between cognition and balance with age.

### Strengths and Limitations

The availability of longitudinal data in NSHD is a major strength of this paper. Ascertainment of multiple cognitive measures at age 53, repeated measures of balance performance, and a range of prospectively ascertained covariates enabled the role of different cognitive processes to be examined, changes with age to be investigated, and allowed exploration of factors that could confound or mediate the associations. However, there are several limitations that should be considered. First, previous NSHD analyses have shown that individuals with missing data or those lost to follow-up have lower SEP across life and are more likely than those with complete data to participate in unhealthy behaviors and be in poor health (20,21). Thus, associations may be underestimated as the analytical sample may include those with higher levels of cognition and balance. This selection effect would be expected to increase with age as more participants are lost to follow-up. This could therefore partially explain why associations weakened slightly with increasing age. However, several analytical steps were taken to minimize potential bias introduced by loss to follow-up. This included the addition of death and attrition indicators, zero imputation of those unable to complete the balance test due to health reasons, and sensitivity analyses in the maximal available sample. Despite using established tests for each cognitive measure, it can be challenging to isolate single cognitive processes within a test. However, the differing associations in mutually adjusted models suggest that distinct facets of each process were appropriately captured within each test. Finally, repeated assessment of balance performance throughout midlife is a major strength of the study; however, there are limitations to using the one-legged stand test. The range of balance scores was limited by floor effects, suggesting the test did not capture the full range of functional ability of the sample. More sensitive assessments of balance and posture, using posturography, may allow better understanding of associations between cognitive processes and balance in future studies.

## Conclusions and Implications

This study has shown evidence of associations of word memory, search speed, verbal fluency, and, to a weaker extent, crystallized cognitive ability with one-legged balance performance that were not explained by other covariates or mutual adjustment of all cognitive measures. Research has begun to acknowledge the interactive nature of cognitive and physical domains in the aging process. Much of this research has focused on aggregate measures of physical and cognitive capabilities; however, it is important to consider individual mechanisms of associations. Given the evidence of independent cognitive mechanisms involved in balance performance, further research should consider how distinct areas of cognitive aging may impact specific functional outcomes such as balance.

These findings could have widespread benefits across several areas of research including: understanding of biological mechanisms, identification of cognitive and physical impairments, and organization of targeted interventions. Further research is needed to assess the potential value of interventions that target specific cognitive exercises in addition to a physical training program (60,61). However, our findings would suggest that this training could target multiple cognitive processes, including but not limited to memory, attention, processing speed, and executive function. Understanding that lower cognitive performance in midlife may be a risk factor for subsequent poor balance ability could also help identify at-risk individuals providing further opportunities for intervention.

## Supplementary Material

glab168_suppl_Supplementary_Tables
