# Supplementary material for: Associations of Word Memory, Verbal Fluency, Processing Speed, and Crystallized Cognitive Ability With One-Legged Balance Performance in Mid- and Later Life
Source: J Gerontol A Biol Sci Med Sci. 2021 Jun 14;77(4):807–16. doi: 10.1093/gerona/glab168 (PMC8974350; doi:10.1093/gerona/glab168)
Supplement: glab168_suppl_Supplementary_Tables [file glab168_suppl_supplementary_tables.docx]

| **eTable 1**. Descriptive characteristics of the analytical sample (n=2934) at age 53 | | | | | |
| --- | --- | --- | --- | --- | --- |
|  | **Men**  (n=1434) | | **Women**  (n=1500) | Tests of sex differences  (p-value) |  |
| **HEALTH STATUS,** n (%) | | |  |  |  |
| **Diabetes** | 44 (3.1) | | 40 (2.7) | 0.51 |  |
| **Knee pain** | 222 (15.6) | | 304 (20.5) | <0.005 |  |
| **Cardiovascular events** | 81 (5.7%) | | 46 (3.1) | <0.005 |  |
| **Respiratory symptoms** | 283 (19.8) | | 274 (18.3) | 0.31 |  |
| **Symptoms of anxiety/depression,** mean (SD) | | 2.6 (0.5) | 2.8 (0.5) | <0.001 |  |
| **BEHAVIOURAL RISK FACTORS**, n (%) | | |  |  |  |
| **Leisure time physical activity** |  | |  |  |  |
| None  1-4 times/month  5+ times/month | 681 (47.5)  267 (18.6)  485 (33.9) | | 757 (50.5)  243 (16.2)  500 (33.3) | 0.18 |  |
| **Smoking status** |  | |  |  |  |
| Current  Previous smoker  Never smoker | 338 (23.6)  730 (50.9)  366 (25.5) | | 338(22.5)  669 (44.6)  493 (32.9) | <0.001 |  |
| **ANTHROPOMETRY,** mean (SD) | | |  |  |  |
| **Height** (m) | 1.75 (0.07) | | 1.62 (0.06) | <0.001 |  |
| **BMI** (kg/m^2^) | 27.4 (4.0) | | 27.4 (5.5) | 0.92 |  |
| **SOCIOECONOMIC INDICATORS**, n (%) | | |  |  |  |
| **Highest household occupational class** | | |  |  |  |
| I Professional/II Intermediate  III Skilled (non-manual or manual)  IV Partly skilled/V Unskilled | 733 (51.4)  539 (37.8)  153 (10.7) | | 534 (35.8)  640 (42.9)  317 (21.3) | <0.001 |  |
| **Educational attainment at age 26** | | |  |  |  |
| Degree or higher  GCE A level or Burnham B  GCE O level or Burnham C  Sub GCE  None attempted | 202 (14.9)  380 (28.0)  200 (14.7)  81 (6.0)  495 (36.5) | | 73 (5.2)  334 (23.6)  366 (25.8)  126 (8.9)  518 (36.6) | <0.001 |  |

| eTable 2. Random-effects models assessing longitudinal associations of cognitive tests at age 53 with log-transformed balance time (ln(sec)) at ages 53 to 69 in i) maximal-available samples and ii) modified fully-adjusted models | | | | | | | | | |
| --- | --- | --- | --- | --- | --- | --- | --- | --- | --- |
|  | | **Change in association per 1 year** | | **Mean % difference in balance time (s) per 1 SD change in cognition at:** | | | | | |
|  |  |  |  | **Age 53** | | **Age 60-64** | | **Age 69** | |
|  |  | Age*cognition coefficient (95% CI) | p-value | Coefficient  (95% CI) | p-value | Coefficient  (95% CI) | p-value | Coefficient  (95% CI) | p-value |
| **i) SEX-ADJUSTED MODELS 1-4 ^a^**  (in maximal available samples) |  | | |  | |  |  |  |  |
| 1. Word memory n=2870 (6851) | | -0.36 (-0.58, -0.14) | *<0.005* | 14.2 (11.6, 16.7) | *<0.001* | 10.6 (8.6, 12.5) | *<0.001* | 8.4 (5.8, 11.0) | <0.001 |
| 2. Search speed n=2912 (6922) | | -0.26 (-0.48, -0.05) | *0.02* | 8.5 (6.0, 11.1) | *<0.001* | 5.9 (3.9, 7.9) | *<0.001* | 4.3 (1.7, 6.9) | <0.005 |
| 3. Verbal fluency n=2927 (6945) | | -0.41 (-0.62, -0.20) | *<0.001* | 12.5 (9.9, 15.0) | *<0.001* | 8.4 (6.4, 10.3) | *<0.001* | 5.9 (3.4, 8.5) | <0.001 |
| 4. NART Linear term  Quadratic term | | -0.41 (-0.62, -0.19)  - ^a^ | *<0.001*  *-* | 15 (12, 18)  2.2 (0.6, 3.7) | *<0.001*  *<0.01* | 11.0 (8.8, 13.2) | *<0.001* | 8.5 (5.7, 11.3) | <0.001 |
|  |  |  |  |  |  | *Estimate constant ^b^* | | | |
| **ii) MODIFIED FULLY-ADJUSTED MODELS**  n=2934 (5466) | |  |  |  |  |  |  |  |  |
| ***A. No age interaction terms*** | |  |  |  |  |  |  |  |  |
| Word memory | | - | *-* | 3.2 (0.7, 5.7) | *0.01* | *Estimate constant ^b^* | | | |
| Search speed | | - | *-* | 2.2 (0.2, 4.1) | *0.03* | *Estimate constant ^b^* | | | |
| Verbal fluency | | - | *-* | 2.1 (-0.1, 4.2) | *0.06* | *Estimate constant ^b^* | | | |
| NART Linear term  Quadratic term | | - | *-* | 1.6 (-1.3, 4.6)  0.5 (-1.2, 2.2) | *0.28*  *0.56* | *Estimate constant ^b^* | | | |
|  |  |  |  |  |  | *Estimate constant ^b^* | | | |
| ***B. Word memory*age only*** | |  |  |  |  |  |  |  |  |
| Word memory | | -0.31 (-0.58, -0.04) | 0.03 | 5.5 (2.3, 8.7) | *<0.005* | 2.4 (-0.1, 5.0) | *0.06* | *0.6 (-2.8, 3.9)* | *0.74* |
| Search speed | | - | *-* | 2.1 (0.2, 4.1) | *0.03* | *Estimate constant ^b^* | | | |
| Verbal fluency | | - | *-* | 2.1 (-0.1, 4.2) | *0.06* | *Estimate constant ^b^* | | | |
| NART Linear term  Quadratic term | | - | *-* | 1.7 (-1.3, 4.6)  0.6 (-1.1, 2.2) | *0.27*  *0.51* | *Estimate constant ^b^* | | | |
|  |  |  |  |  |  | *Estimate constant ^b^* | | | |
| ***C. NART*age only*** | |  |  |  |  |  |  |  |  |
| Word memory | | - | *-* | 3.2 (0.7, 5.7) | *0.01* | *Estimate constant ^b^* | | | |
| Search speed | | - | *-* | 2.2 (0.2, 4.2) | *0.03* | *Estimate constant ^b^* | | | |
| Verbal fluency | | - | *-* | 2.1 (-0.1, 4.2) | *0.06* | *Estimate constant ^b^* | | | |
| NART Linear term  Quadratic term | | -0.31 (-0.61, -0.02)  - | 0.04 | 4.0 (0.3, 7.7)  0.6 (-1.1, 2.3) | *0.03*  *0.47* | *0.9 (-2.2, 3.9)* | *0.58* | *-1.0 (-4.9, 2.9)* | *0.61* |
|  |  |  |  |  |  | *Estimate constant ^b^* | | | |
| *^a^ Interaction term for age*NART^2^ (p=0.59) was not significant and thus removed from the model*  *^b^ Estimates at ages 60-64 and 69 are equivalent to those estimated at age 53* | | | | | | | | | |
